# Concurrence between Guillain-Barré syndrome and immune thrombocytopenic purpura possibly induced by long COVID-19

**DOI:** 10.17843/rpmesp.2022.391.10687

**Published:** 2022-03-31

**Authors:** Rafael Pichardo-Rodríguez, Juan-Jesús Bracamonte-Hernández, Diana-Cristina Ramírez-Meyhuay, Ingrid-Roxani Arquinigo-Lavado, Willy Peña-Oscuvilca, Marcos Saavedra-Velasco, Oscar Ruiz-Franco

**Affiliations:** 1 Instituto de Investigaciones en Ciencias Biomédicas (INICIB), Universidad Ricardo Palma, Lima, Peru. Universidad Ricardo Palma Instituto de Investigaciones en Ciencias Biomédicas (INICIB) Universidad Ricardo Palma Lima Peru; 2 Facultad de Medicina, Universidad Nacional Mayor de San Marcos, Lima, Peru. Universidad Nacional Mayor de San Marcos Facultad de Medicina Universidad Nacional Mayor de San Marcos Lima Peru

**Keywords:** Guillain-Barre Syndrome, Purpura Thrombocytopenic Idiopathic, COVID-19, autoimmunity, Autoimmune diseases, Autoimmune Diseases of the Nervous System, Autoimmune Demyelinating Diseases CNS, Blood Platelet Disorders, thrombocytopenia, SARS-CoV-2

## Abstract

During acute SARS-CoV-2 infection, there is persistent deregulation of the immune system that can last up to 8 months after the acute condition is controlled. This, added to other factors, is possibly associated with an increased risk of the appearance and concurrence of autoimmune diseases. The simultaneous occurrence of GBS and ITP has been rarely reported in the literature, and GBS is rarely associated with another autoimmune disease. We present the case of a man who, one month after his recovery from acute moderate COVID-19, presented concurrent GBS and ITP with an adequate response to treatment.

## INTRODUCTION

During acute infection with severe acute respiratory syndrome type 2 coronavirus (SARVS-CoV-2), there is a persistent dysregulation of the immune system that can last up to eight months after the acute illness has been controlled ^1^. During this period, the patient is apparently more susceptible to developing autoimmune diseases. When we compare the 2019 coronavirus disease (COVID-19) with healthy controls or those infected with other viruses, we find that infection by SARS-CoV-2 is associated with extensive autoantibody production that can cause more than ten autoimmune diseases ^2^.

The nervous system is one of the systems affected by autoimmunity, which can lead to the development of Guillain-Barré syndrome (GBS) ^3^ ^,^ ^4^. It has been suggested that damage to the myelin sheath is possibly due to the antibody production induced by COVID-19, supported by the adequate response to treatment (77.6%) with intravenous immunoglobulin (IV-IG) ^3^ ^,^ ^4^. However, little is known about immune thrombocytopenic purpura (ITP) secondary to COVID-19 (ITP-COVID-19)^ (^ ^5^. Although ITP is more frequently associated with viral infections such as Epstein-Barr virus (EBV), human immunodeficiency virus (HIV), hepatitis C, among others; its association with COVID-19 has also been reported, apparently due to a process of molecular mimicry that generates antiplatelet antibodies ^6^ ^,^ ^7^.

The co-occurrence of GBS and ITP has been rarely reported in the literature, while GBS is rarely associated with another autoimmune disease ^8^. However, persistent dysregulation of the immune system following recovery is possibly key to its development and may be associated with an increased risk of concurrent autoimmune disease.

We present the case of a man who, after recovery from a moderate case of COVID-19, concurrently presented GBS and ITP with adequate response to treatment.

## CASE REPORT

48-year-old male with history of fully treated pulmonary tuberculosis and moderate COVID-19 on 3/21/21, confirmed by real-time polymerase chain reaction that required oxygen therapy administered by binasal cannula due to oxygen saturation of 89%. He did not present hyposmia and received treatment at home with prophylactic anticoagulation and intravenous corticoid therapy for 6 days, 25 days prior to the onset of symptoms.

Onset of symptoms was on 04/15/21 starting with tongue ecchymosis and spontaneous gingival hemorrhage; then he went to a private physician who prescribed mouthwash. Subsequently, petechiae appeared on the upper and lower limbs, which increased in number and size. Then, the patient presented predominantly distal paresthesias in both lower limbs with a burning sensation; he self-medicated several times with diclofenac plus B complex orally. He consulted a private physician again, who requested a brain tomography, which revealed no alterations.

**Figure 1:**
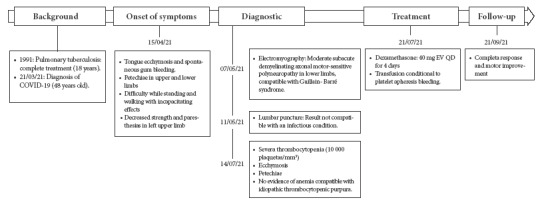
Timeline of the evolution of the patient´s clinical picture: history, onset of symptoms, diagnosis, treatment and follow-up.

Due to the persistence and progression of symptoms (difficulty in standing and walking) and decreased strength and paresthesia in the left upper limb, the patient began physical therapy at home. On 7/05/21 an electromyography showed signs suggestive of moderate subacute axonal motor-sensory demyelinating polyneuropathy in the lower limbs, compatible with GBS. Subsequently, on 11/05/21, due to persistent and progressive disability, he went to the emergency room of a local hospital where he underwent a lumbar puncture that revealed cerebrospinal fluid (CSF) not compatible with an infectious condition (Table 1). The patient requested voluntary discharge and returned home. Four days later, he went to a specialized institute for emergency treatment, where the diagnosis of GBS was confirmed; then he started physiotherapy at home as well as outpatient follow-up. A month and a half later, the patient persisted with ecchymosis and petechiae plus pallor, with severe thrombocytopenia (Table 1); therefore, he was referred to the Dos de Mayo National Hospital on 7/14/21, and admitted through the emergency department (Figure 1).

**Table 1:** Summary of the results of auxiliary laboratory tests

Laboratory tests	Results prior to admission (11/05/21)	Results at admission (07/14/21)	Results two months after discharge (9/16/21)
CSF			
CSF appearance	Slightly cloudy	-	-
CSF cell count	2 cells	-	-
Mononuclear cells in CSF	100%	-	-
Glucose in CSF	85 mg/dL	-	-
Proteins in CSF	27.8 mg/dL	-	-
Germs in CSF	Absent	-	-
Hemoglobin	12 g/dL	-	-
Platelet count	10,000 platelets/mm^3^	30,000 platelets/mm^3^	150,000 platelets/mm^3^
HIV	-	Non-reactive	-
Hepatitis C	-	Non-reactive	-
Hepatitis D	-	Non-reactive	-
Syphilis	-	Non-reactive	-
Direct Coombs test	-	Negative	-
Antinuclear antibodies	-	Negative	-
Extractable nuclear antigen profile	-	Negative	-

CSF: Cerebrospinal fluid. HIV: Human immunodeficiency virus.

Physical examination revealed gingival hemorrhage and ecchymosis in the upper and lower limbs. He also had decreased strength in the left upper limb. During his stay he was evaluated by the hematology department who diagnosed ITP secondary to long COVID-19. The neurology department reevaluated the case and confirmed the diagnosis of GBS, and requested an MRI, which, together with EBV serology, could not be performed due to lack of financial resources.

Treatment started with dexamethasone at 40 mg PO QD h for 4 days, and apheresis platelet transfusion conditional to bleeding. The patient responds adequately with partial response and is discharged with prednisone at 75 mg PO QD.

The patient was reevaluated two months later and showed complete response and motor improvement (Table 1).

## DISCUSSION

During acute SARS-CoV-2 infection there is dysregulation of the immune system associated with lymphopenia and increased expression of inflammatory mediators ^1^. Subsequently, some patients develop physical and neuropsychiatric symptoms that persist for more than twelve weeks, which is called long COVID-19. Apparently, this immune system dysfunction would also persist up to eight months after the acute phase, characterized by depletion of virgin T and B cells and elevated expression of type I interferon, interleukin-6 and type III interferon, which is probably a post-acute sequela of COVID-19 ^1^.

Concurrence between GBS and ITP is rarely reported in the published literature, and GBS is very infrequently associated with another autoimmune disease ^8^. In the presented case, the concurrence of these two diseases occurred 25 days after acute infection and possibly due to the persistent immune dysregulation of long COVID-19. According to reports, only 26.6% of patients with GBS secondary to COVID-19 (GBS-COVID-19) developed it 21 days after symptom onset, on the other hand, ITP-COVID-19 can develop up to 30 days after symptom onset ^5^ ^,^ ^9^. Adequate follow-up of patients recover from acute illness is important.

Possible immunopathological mechanisms include the virus stimulating production by a mechanism of molecular mimicry with cross-reaction to certain glycoproteins of the platelet surface, leading to its destruction by the reticuloendothelial system ^5^. Additionally, these antibodies could inhibit the development of megakaryocytes, promoting their apoptosis in the bone marrow which reduces platelet production ^5^. Epitope dissemination caused by the release of autoantigens after virus-induced tissue damage is probably involved ^5^. On the other hand, the virus could trigger an adaptive immune response in the nervous system, where interactions between T and B cells could result in the production of specific antibodies similar to the ganglioside peptide structure, leading to loss of self-tolerance ^10^. Gangliosides on the membranes of neurons and Schwann cells act as receptors for anti-ganglioside antibodies, becoming targets for the destruction of their myelin sheaths or axons ^10^.

Furthermore, it adds to the reactivation of latent viruses, such as EBV which is found in 66.7% of prolonged COVID cases ^11^. This further increases the risk of developing autoimmunity since EBV leads to the development of autoimmune diseases ^12^. In our case, its presence could not be determined due to lack of resources.

The most frequent electromyographic findings in GBS-COVID-19 include demyelination (62.9%), followed by sural sparing associated with demyelination (10%), mixed demyelination and axonal damage (7.1%), among others. We observed demyelination and decreased limb strength, which is the most frequent clinical manifestation (75.4%) of GBS-COVID-19. Likewise, proteins in CSF samples are reported to range between 40 and 193 mg/ dL, elevated glycorrhachia is also reported; in our patient, CSF proteins were less than the lower limit, with glycorrhachia within the normal range ^3^ ^,^ ^13^. On the other hand, thrombocytopenia is a complication of COVID-19 and is directly related to the severity of the disease ^6^. Platelet count in our patient was close to the median (5000 platelets/mm^3^) observed in patients with ITP-COVID-19, as well as the presence of petechiae and ecchymosis at the time of diagnosis, which are the most frequent manifestations ^5^.

The patient received treatment for ITP according to the recommendations for corticosteroid-based management during the acute phase ^14^. In cases of ITP-COVID-19, both the use of IV-IG, as well as corticosteroids and their combinations, apparently achieve a complete response rate and partial response in 85% of patients ^5^. The patient had an adequate response, achieving a complete response after two months of treatment. Plasmapheresis and IV-IG were not required for the management of GBS, since there was an adequate response with corticoid and physical therapy. Apparently, response rates in patients with GBS-COVID-19 are not high (28%) as observed in ITP-COVID-19 cases, based mainly on management with IV-IG, plasmapheresis and corticoids ^10^. More than 70% of patients have good prognosis ^15^.

In conclusion, concurrence between GBS and ITP is unlikely, however, it could occur secondary to persistent immune system dysfunction caused by long COVID-19. We recommend close follow-up of patients recovering from the acute phase, as well as identification of predictors for the development of autoimmune disorders.
