# Supplementary material for: Concurrence between Guillain-Barré syndrome and immune thrombocytopenic purpura possibly induced by long COVID-19
Source: Rev Peru Med Exp Salud Publica. 2022 Mar 31;39(1):111–4. doi: 10.17843/rpmesp.2022.391.10687 (PMC11397667; doi:10.17843/rpmesp.2022.391.10687)
Supplement: Translation

# Translation: Concurrencia entre el síndrome de Guillain-Barré y púrpura trombocitopénica inmune posiblemente inducidos por COVID-19 prolongado

**Authors:** Rafael Pichardo-Rodríguez, Juan-Jesús Bracamonte-Hernández, Diana-Cristina Ramírez-Meyhuay, Ingrid-Roxani Arquinigo-Lavado, Willy Peña-Oscuvilca, Marcos Saavedra-Velasco, Oscar Ruiz-Franco

**Affiliations:** 1 Instituto de Investigaciones en Ciencias Biomédicas (INICIB), Universidad Ricardo Palma, Lima, Perú.; 2 Facultad de Medicina, Universidad Nacional Mayor de San Marcos, Lima, Perú.

**Keywords:** Síndrome de Guillain-Barré, Púrpura Trombocitopénica Idiopática, COVID-19, Autoinmunidad, Enfermedades Autoinmunes, Enfermedades Autoinmunes del Sistema Nervioso, Enfermedades Autoinmunes Desmielinizantes SNC, Trastornos de las Plaquetas Sanguíneas, Trombocitopenia, SARS-CoV-2

## Abstract

Durante la infección aguda por el SARVS-CoV-2 se produce una desregulación del sistema inmune que puede durar hasta ocho meses después de controlado el cuadro agudo. Esto, sumado a otros factores, posiblemente este asociado con un aumento del riesgo de aparición y concurrencia de enfermedades autoinmunes. La aparición simultanea del síndrome de Guillain-Barré (SGB) y púrpura trombocitopénica (PTI) se ha reportado poco en la literatura, y el SGB raramente se asocia con otra enfermedad autoinmune. Presentamos el caso de un varón que luego de un mes de tener un cuadro agudo de COVID-19 moderado, presentó concurrentemente SGB y PTI con respuesta adecuada al tratamiento.

## INTRODUCCIÓN

Durante la infección aguda por el coronavirus de tipo 2 causante del síndrome respiratorio agudo severo (SARVS-CoV-2), se produce una desregulación persistente del sistema inmune que puede durar hasta ocho meses después de controlado el cuadro agudo ^1^. Durante este período, aparentemente el paciente es más susceptible a desarrollar enfermedades autoinmunes. Cuando comparamos la enfermedad por coronavirus del 2019 (COVID-19) con controles sanos o con aquellos infectados por otros virus, la infección por SARS-CoV-2 se asocia con una extensa generación de autoanticuerpos que pueden originar más de diez enfermedades autoinmunes ^2^. 

Uno de los sistemas afectados por esta autoinmunidad es el sistema nervioso, que conlleva al desarrollo del síndrome de Guillain-Barré (SGB) ^3^ ^,^ ^4^. Se postula que el daño en la vaina de mielina se deba, posiblemente, a la producción de anticuerpos inducida por la COVID-19, apoyándose en la adecuada respuesta al tratamiento (77,6%) con inmunoglobulina intravenosa (IG-IV) ^3^ ^,^ ^4^. Sin embargo, es poco lo que se conoce acerca de la púrpura trombocitopénica inmune (PTI) secundaria a la COVID-19 (PTI-COVID-19) ^5^. Si bien la PTI se encuentra asociada con mayor frecuencia a infecciones virales como el virus de Epstein-Barr (VEB), el virus de la inmunodeficiencia humana (VIH), la hepatitis C, entre otros; también se ha reportado su asociación con la COVID-19, aparentemente debido a un proceso de mimetismo molecular que genera anticuerpos antiplaquetarios ^6^ ^,^ ^7^.

La aparición simultanea del SGB y PTI se ha reportado poco en la literatura, en tanto que el SGB raramente se asocia con otra enfermedad autoinmune ^8^. Sin embargo, la desregulación persistente del sistema inmune, posterior a la recuperación, posiblemente sea clave para su desarrollo y se asocie con un aumento del riesgo de concurrencia de enfermedades autoinmunes.

Se presenta el caso de un varón que, luego de su recuperación de un cuadro moderado de COVID-19 presentó concurrentemente SGB y PTI con respuesta adecuada al tratamiento.

## REPORTE DE CASO

Varón de 48 años con antecedente de tuberculosis pulmonar con tratamiento completo y COVID-19 moderado el 21/03/21, confirmado mediante reacción en cadena de la polimerasa en tiempo real con requerimiento de oxígeno con cánula binasal por saturación de oxígeno en 89%, sin hiposmia con manejo domiciliario con anticoagulación profiláctica y corticoterapia endovenosa por 6 días, 25 días previos al inicio de los síntomas. 

El paciente inicia su enfermedad el 15/04/21 con equimosis en lengua y gingivorragia espontánea; acude a un médico particular quien le indica enjuagues bucales. Posteriormente aparecen petequias en miembros superiores e inferiores que aumentaron en número y tamaño. Luego, presenta parestesias a predominio distal de ambos miembros inferiores con sensación de quemazón; se automedica, en diversas ocasiones, con diclofenaco más complejo B por vía oral. Acude nuevamente a un médico particular, quien ordena una tomografía multicorte cerebral, la cual no reveló alteraciones.

Por la persistencia y progresión de los síntomas (dificultad para la bipedestación y marcha) y disminución de fuerza y parestesias en miembro superior izquierdo, el paciente inicia fisioterapias en casa. El 7/05/21 se le realiza una electromiografía en la que se encuentran signos sugerentes de polineuropatía subaguda desmielinizante axonal motora-sensitivo, en miembros inferiores, de grado moderado, compatible con SGB. Posteriormente, el 11/05/21, debido a la incapacidad persistente y progresiva, acude a emergencias de un hospital local donde le realizan una punción lumbar que evidencia un líquido cefalorraquídeo (LCR) no compatible con un cuadro infeccioso (Tabla 1). El paciente solicita su alta voluntaria y retorna a su domicilio. Cuatro días después, acude por emergencia a un instituto especializado donde se confirma el diagnóstico de SGB, y se inicia fisioterapia en domicilio y control por consultorio externo. Un mes y medio después, el paciente persiste con equimosis y petequias más palidez con plaquetopenia severa (Tabla 1); por lo cual es referido al Hospital Nacional Dos de Mayo el 14/07/21, e ingresa por el servicio de emergencia (Figura 1).

**Tabla 1:** Resumen de los resultados de los exámenes auxiliares laboratorio

Exámenes de laboratorio	Resultados previos al ingreso (11/05/21)	Resultados al ingreso (14/07/21)	Resultados dos meses después del alta (16/09/21)
LCR			
Aspecto LCR	Ligeramente turbio	-	-
Recuento celular en LCR	2 células	-	-
Mononucleares en LCR	100%	-	-
Glucosa en LCR	85 mg/dL	-	-
Proteínas en LCR	27,8 mg/dL	-	-
Gérmenes en LCR	Ausentes	-	-
Hemoglobina	12 g/dL	-	-
Recuento plaquetario	10 000 plaquetas/mm^3^	30 000 plaquetas/mm^3^	158 000 plaquetas/mm^3^
VIH	-	No reactivo	-
Hepatitis C	-	No reactivo	-
Hepatitis B	-	No reactivo	-
Sífilis	-	No reactivo	-
Prueba de Coombs directo	-	Negativo	-
Anticuerpos antinucleares	-	Negativo	-
Perfil de antígeno nuclear extraíble	-	Negativo	-

LCR: líquido cefalorraquídeo. VIH: virus de la inmunodeficiencia humana.

**Figura 1:** Línea de tiempo de la evolución del cuadro clínico del paciente; antecedentes, inicio de enfermedad, diagnóstico, tratamiento y seguimiento.

Al examen físico se evidencia gingivorragia y equimosis en miembros superiores e inferiores. Asimismo, tenía disminución de la fuerza en miembro superior izquierdo. Durante su estancia es evaluado en el servicio de hematología y se plantea el diagnóstico de PTI secundario a COVID-19 prolongado. El servicio de neurología reevalúa el caso y confirma el diagnóstico de SGB, solicita una resonancia magnética que, junto con la serología para VEB, no se pudo realizar por falta de recursos económicos. 

Se inicia tratamiento con dexametasona a 40 mg EV c/24 h durante 4 días, y transfusión de aféresis de plaquetas condicional a sangrado. El paciente responde adecuadamente con respuesta parcial y es dado de alta con prednisona a 75 mg VO c/24 h.

El paciente es reevaluado dos meses después donde se observa que presenta respuesta completa y mejoría a nivel motor (Tabla 1).

## DISCUSIÓN

Durante la infección aguda por el SARS-CoV-2 hay una desregulación del sistema inmune asociada con linfopenia y aumento de la expresión de mediadores inflamatorios ^1^. Posteriormente, algunos pacientes desarrollan síntomas físicos y neuropsiquiátricos que persisten más de doce semanas denominado COVID-19 prolongado. Aparentemente esta disfunción del sistema inmune también persistiría hasta ocho meses después del cuadro agudo, caracterizado por depleción de las células T y B vírgenes y una elevada expresión de interferón tipo I, interleucina-6 e interferón tipo III que, probablemente, sea un secuela posaguda de la COVID-19 ^1^. 

La concurrencia entre el SGB y PTI ha sido raramente publicada en la literatura, y el SGB con muy poca frecuencia se asocia con otra enfermedad autoinmune ^8^. En el caso presentado, la concurrencia de estas dos enfermedades se dio 25 días después de la infección aguda y posiblemente por causa de la desregulación inmunológica persistente del COVID-19 prolongado. Según los reportes, solo el 26,6% de los pacientes con SGB secundario a COVID-19 (SGB-COVID-19) lo desarrolló 21 días posteriores al inicio de síntomas y, por otro lado, la PTI-COVID-19 puede desarrollarse hasta 30 días después del inicio de síntomas ^5^ ^,^ ^9^. Es importante realizar un seguimiento adecuado a los pacientes recuperados del cuadro agudo.

Entre los posibles mecanismos inmunopatológicos, el virus estimularía la producción por un mecanismo de mimetismo molecular con reacción cruzada a ciertas glicoproteínas de la superficie plaquetaria conllevando a su destrucción por el sistema reticuloendotelial ^5^. Adicionalmente, estos anticuerpos inhibirían el desarrollo de los megacariocitos, promoviendo su apoptosis en la médula ósea lo cual reduce la producción de plaquetas ^5^. Probablemente la propagación de epítopos causado por la liberación de autoantígenos después del daño tisular inducido por el virus esté implicada ^5^. Por otro lado, en el sistema nervioso, el virus desencadenaría una respuesta inmunitaria adaptativa, donde las interacciones entre las células T y B darían como resultado la producción de anticuerpos específicos similares a la estructura del péptido gangliósido, lo que lleva a la pérdida de autotolerancia ^10^. Los gangliósidos de las membranas de las neuronas y las células de Schwann actúan como receptores de los anticuerpos antigangliósidos, convirtiéndose en objetivos para la destrucción de las vainas de mielina o axones ^10^. 

Por otro lado, se suma a la reactivación de virus latentes, como el VEB que se observa en el 66,7% de los casos de COVID prolongado ^11^. Esto incrementa aun más el riesgo de desarrollar autoinmunidad ya que el VEB conduce al desarrollo de enfermedades autoinmunes ^12^. En nuestro caso no se pudo determinar su presencia por falta de recursos. 

Entre los hallazgos electromiográficos más frecuentes en el SGB-COVID-19 se encuentra la desmielinización (62,9%), seguido de preservación sural asociada con desmielinización (10%), desmielinización mixta y daño axonal (7,1%), entre otros. Nosotros observamos desmielinización y diminución de la fuerza en las extremidades, que es la manifestación clínica más frecuente (75,4%) del SGB-COVID-19. Asimismo, se reporta que la proteinorraquia en las muestras de LCR oscilan entre 40 y 193 mg/dL, así como, glucorraquia elevada; en nuestro paciente se observó proteinorraquia menor del límite inferior con glucorraquia dentro de los rangos normales ^3^ ^,^ ^13^. Por otro lado, la trombocitopenia es una complicación de la COVID-19 y se encuentra directamente relacionada con la severidad de la enfermedad ^6^. El recuento plaquetario en nuestro paciente estuvo cercano a la mediana (5000 plaquetas/mm^3^) observada en los pacientes con PTI-COVID-19, así como la presencia de petequias y equimosis al momento del diagnóstico, que son las manifestaciones más frecuentes ^5^.

El paciente recibió tratamiento para la PTI de acuerdo a las recomendaciones para el manejo de la fase aguda basado en corticoides ^14^. En los casos de PTI-COVID-19, tanto el uso de IG-IV, así como corticoides y sus combinaciones, aparentemente logran alcanzar una tasa de respuesta completa y respuesta parcial en el 85% de los pacientes ^5^. El paciente tuvo una respuesta adecuada, alcanzando una respuesta completa a los dos meses de tratamiento. Para el manejo del SGB no se requirió el uso de plasmaféresis ni IG-IV, pues con la corticoterapia y terapia física se obtuvo una respuesta adecuada. Aparentemente la tasas de respuesta en los pacientes con SGB-COVID-19 no son altas (28%) como se observa en la PTI-COVID-19, basándose el manejo en IG-IV, plasmaféresis y corticoides, principalmente ^10^. Más del 70% de los pacientes presentan un buen pronóstico ^15^.

En conclusión, la concurrencia entre el SGB y PTI es poco probable, sin embargo, podría darse secundario a una disfunción persistente del sistema inmune causado por el COVID-19 prolongado. Se recomienda realizar un seguimiento estrecho a los pacientes que se recuperan de la fase aguda y determinar predictores para el desarrollo de trastornos autoinmunes.
